# Mitral and tricuspid valve surgery in an adult with aberrant left brachiocephalic vein: a case report

**DOI:** 10.1093/jscr/rjaf689

**Published:** 2025-09-03

**Authors:** Masafumi Kudo, Hideki Tsubota, Yuki Akaguma, Masanori Honda, Hitoshi Okabayashi

**Affiliations:** Department of Cardiovascular Surgery, Mitsubishi Kyoto Hospital, 1 Katsuragosho-cho, Nishikyo-ku, Kyoto 615-8087, Japan; Department of Cardiovascular Surgery, Mitsubishi Kyoto Hospital, 1 Katsuragosho-cho, Nishikyo-ku, Kyoto 615-8087, Japan; Department of Cardiovascular Surgery, Mitsubishi Kyoto Hospital, 1 Katsuragosho-cho, Nishikyo-ku, Kyoto 615-8087, Japan; Department of Cardiovascular Surgery, Mitsubishi Kyoto Hospital, 1 Katsuragosho-cho, Nishikyo-ku, Kyoto 615-8087, Japan; Department of Cardiovascular Surgery, Mitsubishi Kyoto Hospital, 1 Katsuragosho-cho, Nishikyo-ku, Kyoto 615-8087, Japan

**Keywords:** aberrant brachiocephalic vein, mitral valve, tricuspid valve, superior vena cava, cardiac surgery

## Abstract

Aberrant left brachiocephalic vein (ALBCV), in which the vein passes posterior to the ascending aorta, is a rare vascular anomaly in adults without congenital heart disease. This condition can complicate venous cannulation during cardiac surgery. We report the case of a 78-year-old woman with severe mitral and tricuspid regurgitation. Preoperative imaging revealed an ALBCV joining the superior vena cava near the right atrium. Although the ALBCV was exposed from both sides of the ascending aorta and prepared for potential cannulation, the cardiopulmonary bypass flow rate was maintained using standard bicaval cannulation. Mitral valve replacement, tricuspid annuloplasty, left atrial Maze, and left appendage resection were performed without any complications. Although ALBCV may present challenges during surgery, thorough preoperative recognition and surgical planning allows for successful management. This case underscores the importance of identifying vascular anomalies, such as ALBCV, before cardiac surgery to avoid unexpected complications and ensure optimal outcomes.

## Introduction

Aberrant left brachiocephalic vein (ALBCV), where the vein passes posterior to the ascending aorta, is a rare venous anomaly in adults [1]. It is usually associated with congenital heart disease (CHD) in pediatric patients and is often referred to as a ‘retro-aortic innominate vein’ in the CHD literature [2]. Although usually asymptomatic and incidentally detected on imaging, ALBCV may pose challenges during cardiac surgery owing to potential issues with venous cannulation and drainage during cardiopulmonary bypass (CPB). We report a rare adult case of cardiac surgery for mitral and tricuspid regurgitation with an ALBCV and discuss operative considerations.

## Case report

**Figure 1:**
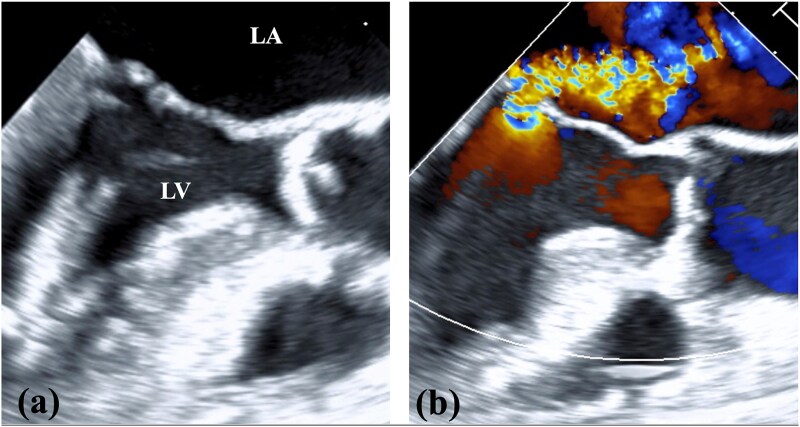
TEE findings. (a) Shortening of the anterior and posterior leaflets of the mitral valve was noted with thickening of the commissure. (b) Both valve leaflets were shortened and the coaptation zone was stiff, resulting in severe MR. LA, left atrium; LV, left ventricle.

**Figure 2:**
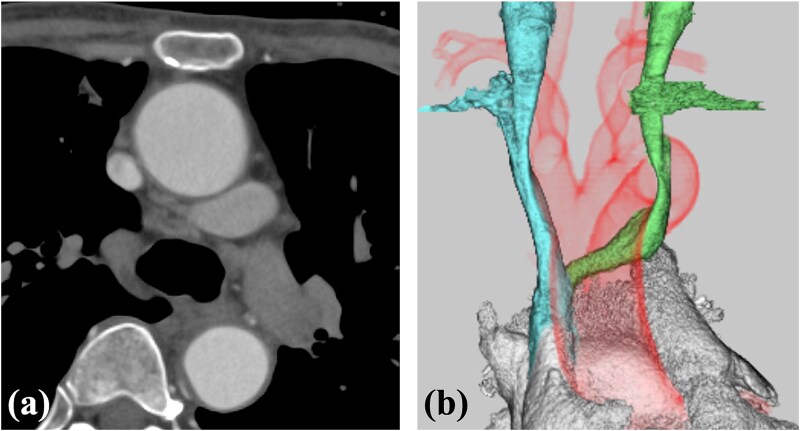
Contrast-enhanced CT. (a) The ALBCV coursed posterior to the ascending aorta. (b) The ALBCV joined the SVC at a peripheral location ⁓3 cm from the right atrium. No right aortic arch or double aortic arch was observed.

**Figure 3:**
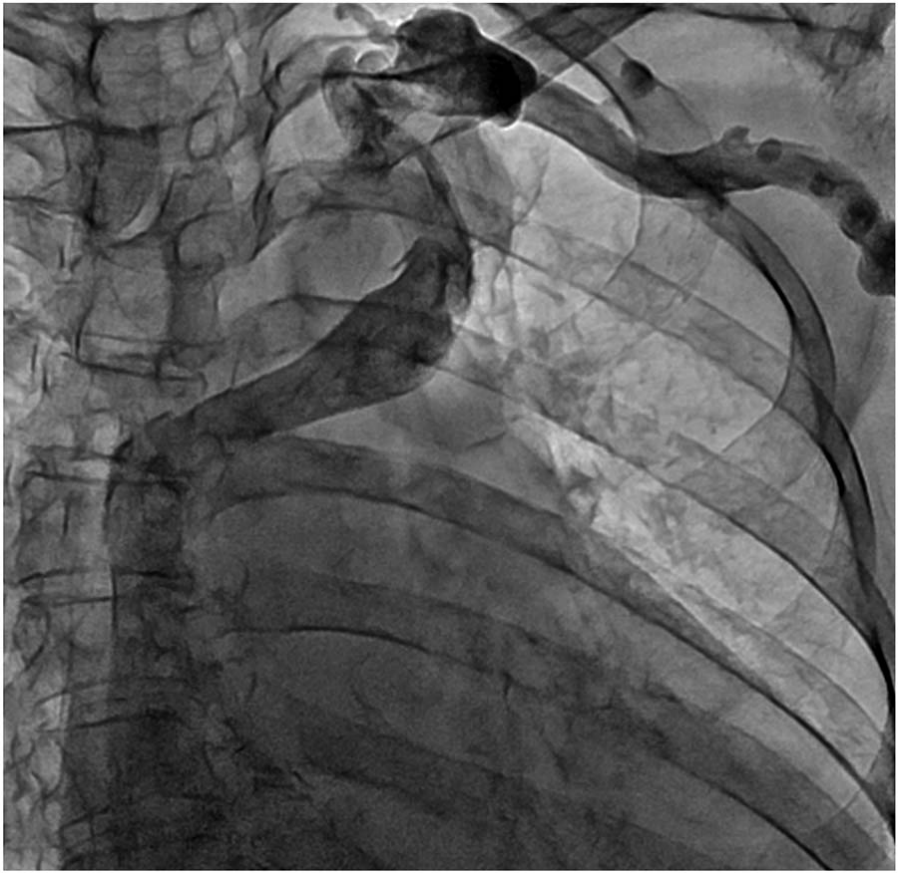
Venography of the left upper limb. The ALBCV joined the SVC, and no communication was observed between the ALBCV and coronary sinus.

A 78-year-old woman with severe mitral regurgitation (MR), tricuspid regurgitation, and atrial fibrillation was admitted with worsening heart failure. She had hypertension, but no known CHD. She was 138 cm tall, weighing 34.6 kg, with a body mass index of 18.3 kg/m^2^. Her vital signs were normal. The N-terminal fragment of pro B-type natriuretic peptide was elevated at 2577 pg/mL. Chest radiography revealed cardiac enlargement and bilateral pulmonary edema. Transesophageal echocardiography (TEE) revealed shortening of the anterior and posterior leaflets of the mitral valve, with thickening of the commissure, indicating severe MR (Fig. 1 and Video  1). The tricuspid annulus diameter increased to 29.4 mm. No congenital heart diseases were detected. Contrast-enhanced computed tomography (CT) revealed that the left innominate vein coursed posterior to the ascending aorta, the so-called ALBCV, and joined the superior vena cava (SVC) 3 cm from the right atrium (Fig. 2 and Video  2). Venography of the left upper limb confirmed the ALBCV (Fig. 3 and Video  3). No communication existed between the ALBCV and the coronary sinus.

**Figure 4:**
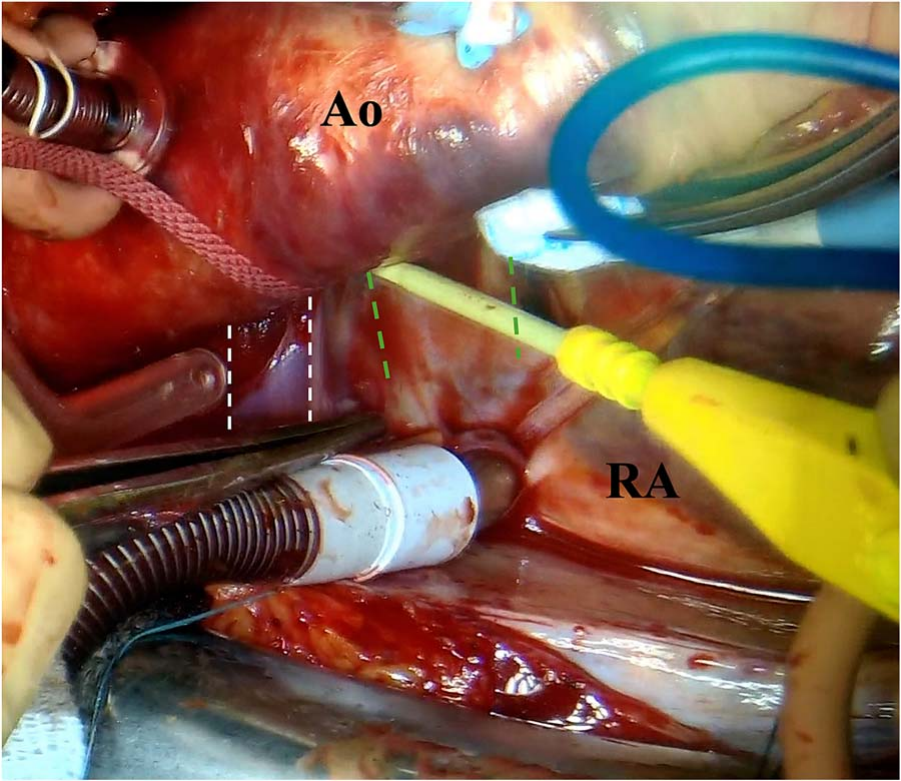
Photograph of the surgeon’s view. The left double-dotted line indicates ALBCV. The right double-dotted line indicates the right pulmonary artery. Ao, aorta; RA, right atrium.

We approached using a median sternotomy and inserted a cannula into the ascending aorta, a 22 Fr right angle cannula directly into the SVC and a 28 Fr straight cannula into the inferior vena cava. The ALBCV was exposed from the left side of the ascending aorta and prepared for potential cannulation. The dorsal side of the ascending aorta was dissected from the right side, confirming ALBCV joined the SVC (Fig. 4 and Video  4). The cannula tip in the SVC did not extend beyond the ALBCV junction. Extracorporeal circulation flow rate was maintained at 2.6 L/min/m^2^, requiring no additional ALBCV cannula.

Vertical left atriotomy and left atrial Maze were performed, followed by left atrial appendage resection. The mitral valve leaflet in P2 and P3 was markedly shortened with lost mobility, and the A2 coaptation zone was thickened and hardened. Mitral valve replacement used the MITRIS RESILIA mitral valve 25 mm (Edwards Lifesciences, Irvine, CA, USA). The right atrium was incised longitudinally. Tricuspid annuloplasty used the SJM Tailor band 27 mm (St. Jude Medical Inc., St. Paul, Minn., USA). Total operative time, CPB time, and aortic cross-clamp time were 255, 147, and 95 min, respectively.

The patient was weaned off the ventilator on surgery day without central nervous system symptoms. She was discharged on the 19th postoperative day and, at 5 months postoperatively, showed no recurrence of mitral or tricuspid regurgitation, with maintained sinus rhythm.

## Discussion

This case demonstrated that although we had carefully planned perioperative strategies for ALBCV, no special intraoperative measures were ultimately required. This outcome likely resulted from the detailed preoperative imaging and strategic flexibility. ALBCV, also called a retro-aortic innominate vein*,* is a rare venous anomaly where the left brachiocephalic vein courses posterior to the ascending aorta [1, 3, 4]. This anomaly commonly occurs in pediatric patients with CHD, such as tetralogy of Fallot, atrial, or ventricular septal defects, right aortic arch, and double aortic arch [5]. Adult cases without CHD are rare, with few documented in literature [6–8]. Kobayashi *et al.* [1] reviewed CT images of 49 494 cases to investigate the incidence of persistent left superior vena cava (PLSVC) and ALBCV. They found PLSVC incidence was 0.15%, and ALBCV was only 0.055%. Although ALBCV is often asymptomatic and discovered incidentally, it may pose risks during central venous catheterization, device placement, and cardiac surgery. The posterior course of the vein can interfere with surgical exposure and complicate venous drainage during CPB, potentially causing inadequate perfusion and increased venous pressure. If the retro-aortic course is not recognized, the vein may be inadvertently injured during aortic cross-clamp placement. When the innominate vein is not visualized after median sternotomy, the differential diagnoses should include a retro-aortic course or PLSVC with an absent bridging vein. Awareness of these possibilities can help prevent inadvertent vascular injuries.

In our case, the anomaly was identified on preoperative contrast-enhanced CT and venography, showing the retroaortic course of the left brachiocephalic vein draining into the SVC, ⁓3 cm from the right atrium. Based on these findings, we considered the possibility of adding a venous cannula to ALBCV. However, satisfactory venous return was achieved with conventional bicaval cannulation alone, without ALBCV cannulation.

This experience highlights two key lessons. First, careful preoperative imaging is essential for detecting vascular anomalies like ALBCV, which may be overlooked. Second, anticipating challenges and planning alternative strategies can help to avoid intraoperative surprises and ensure safe outcomes. In our case, the absence of congenital anomalies and sufficient length and caliber of the ALBCV contributed to successful CPB establishment without additional cannulation. As cardiac imaging becomes more widespread among the aging population, incidental detection of vascular anomalies like ALBCV may become more common. Therefore, cardiac surgeons should be familiar with these variations and incorporate them into preoperative assessments.

This is among the few reports describing successful cardiac surgery in adults with incidentally discovered ALBCV. This case contributes to awareness of this anomaly and emphasizes the importance of preoperative evaluation and surgical planning. In this adult cardiac surgery case with ALBCV, successful surgery was achieved without complications through preoperative evaluation of the anomalous vein course. Accurate preoperative imaging and planning are essential for safe surgical management of this rare vascular anomaly.

## Supplementary Material

Video_1_rjaf689 Additional file 1: Video 1. Preoperative TEE findings. TEE: transesophageal echocardiography.

Video_2_rjaf689 Additional file 2: Video 2. Contrast-enhanced CT findings. CT: computed tomography.

Video_3_rjaf689 Additional file 3: Video 3. Venography of the left upper limb.

Video_4_rjaf689 Additional file 4: Video 4. Operative findings from the surgeon's view.
